# Supplementary material for: Bacteriophage‐Mimetic DNA Origami Needle for Targeted Membrane Penetration and Cytosolic Cargo Delivery
Source: Adv Sci (Weinh). 2026 Jan 9;13(10):e12844. doi: 10.1002/advs.202512844 (PMC12915191; doi:10.1002/advs.202512844)
Supplement: Supplementary file 1 — Supporting File: advs73643‐sup‐0001‐SuppMat.docx. [file ADVS-13-e12844-s001.docx]

***Supplementary information for***

**Bacteriophage-Mimetic DNA Origami Needle for Targeted Membrane Penetration and Cytosolic Cargo Delivery**

***Authors:***

Anirban Samanta^a,b+^, Mette Galsgaard Malle^a^*^+^, Emily Tsang^a,b^, Marjan Omer^a^, Mads K. Skaanning^a,b^, Sarah Youssef^a,b^, Jørgen Kjems^a,c^, Kurt V. Gothelf^a,b^*

*Affiliation:*

*^a^*Interdisciplinary Nanoscience Center (iNANO), Aarhus University, 8000 Aarhus C, Denmark

^b^Department of Chemistry, Aarhus University, 8000 Aarhus C, Denmark

^c^Department of Molecular Biology and Genetics, Aarhus University, 8000 Aarhus C, Denmark

**Figure S1. Blueprint of scaffold and staple routing.**

Illustration of the scaffold and staple routing, as well as the helical arrangement, exported from Cadnano. Helix 0 to Helix 5 makes up the needle tip.


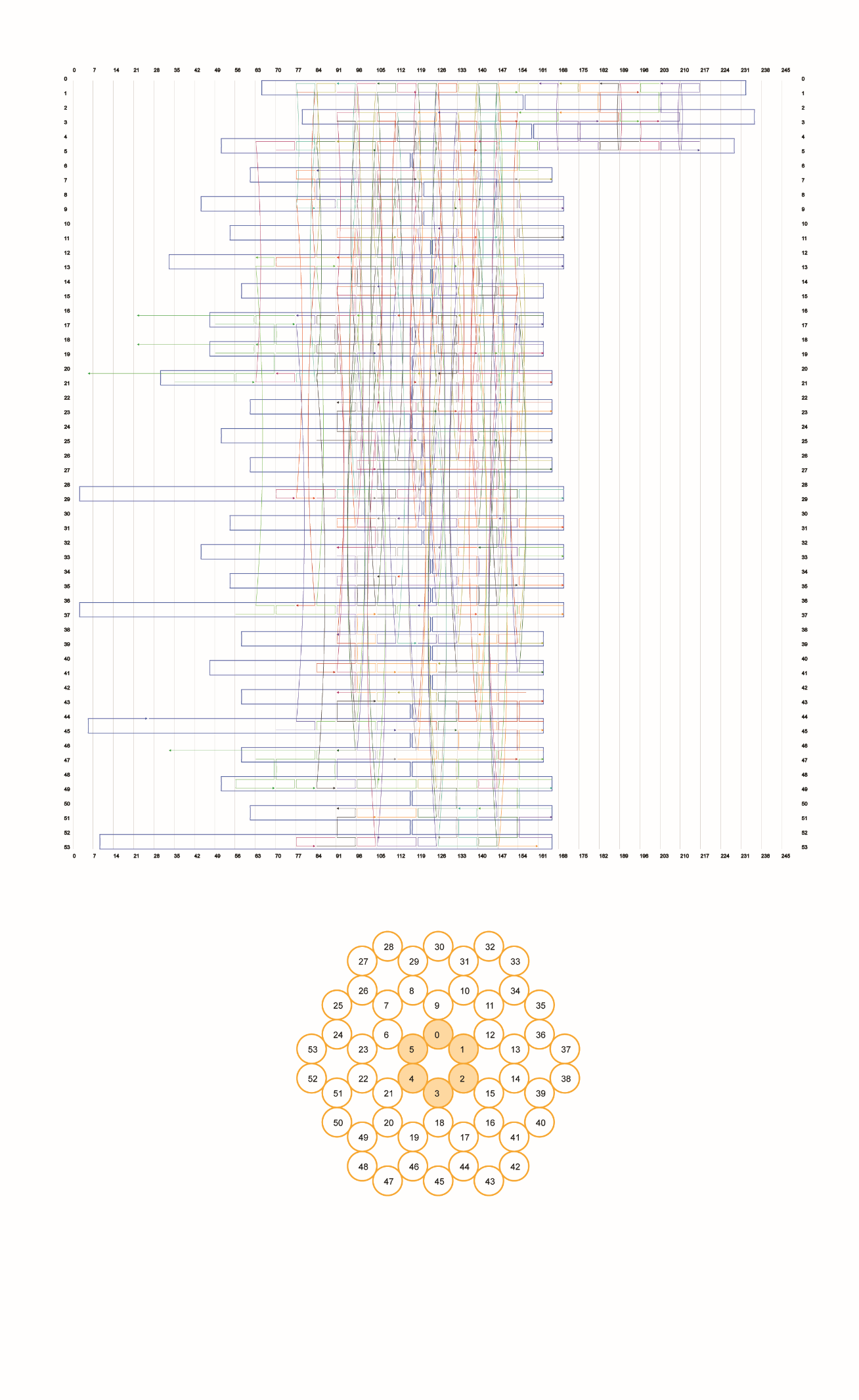


**Figure S2. TEM image of unfunctionalized needle.**

Full TEM image of the purified structure in 20 mM MgCl2, 50 mM NaCl, 1x TAE, 52,000x magnification. Scale bar is 100 nm. A crop-out of this image was used in Fig. 2c.


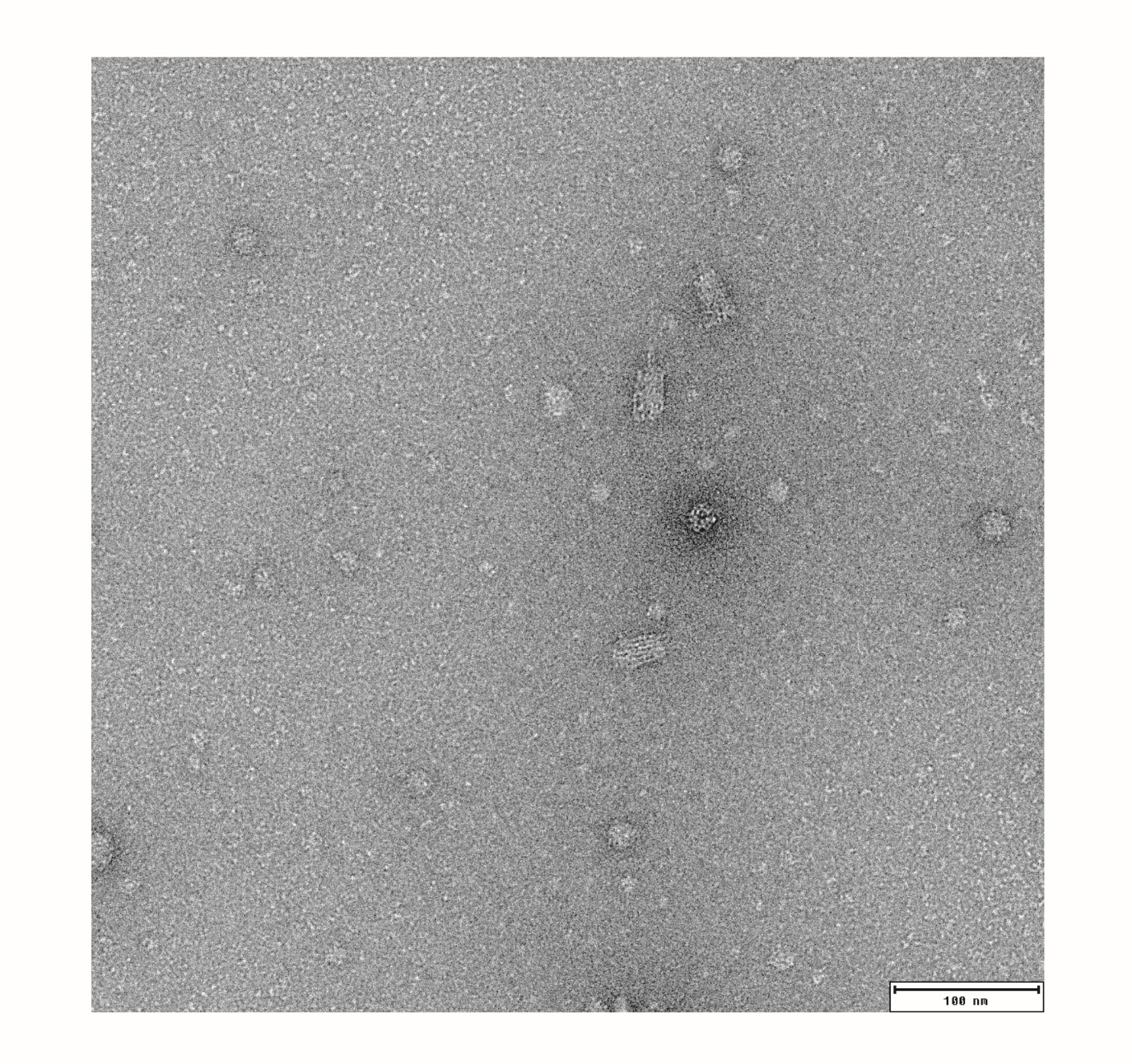


**Figure S3. 3D and simplified illustration of the origami needle design.**

(a) A 3-dimensional rendering of the origami needle design depicturing the estimated dimension of the base structure as 50 nm × 20 nm. b) Schematic of the needle tip of the origami design showing the positions for capturing staple strands extensions for functionalization with both Trastuzumab antibodies and cholesterol. (c) A simplified illustration highlighting the capturing staple for trastuzumab and the cholesterol capture staples, as well as the payload and reporter dye binding configurations. (d) A schematic of the assembly progress of the full needle.


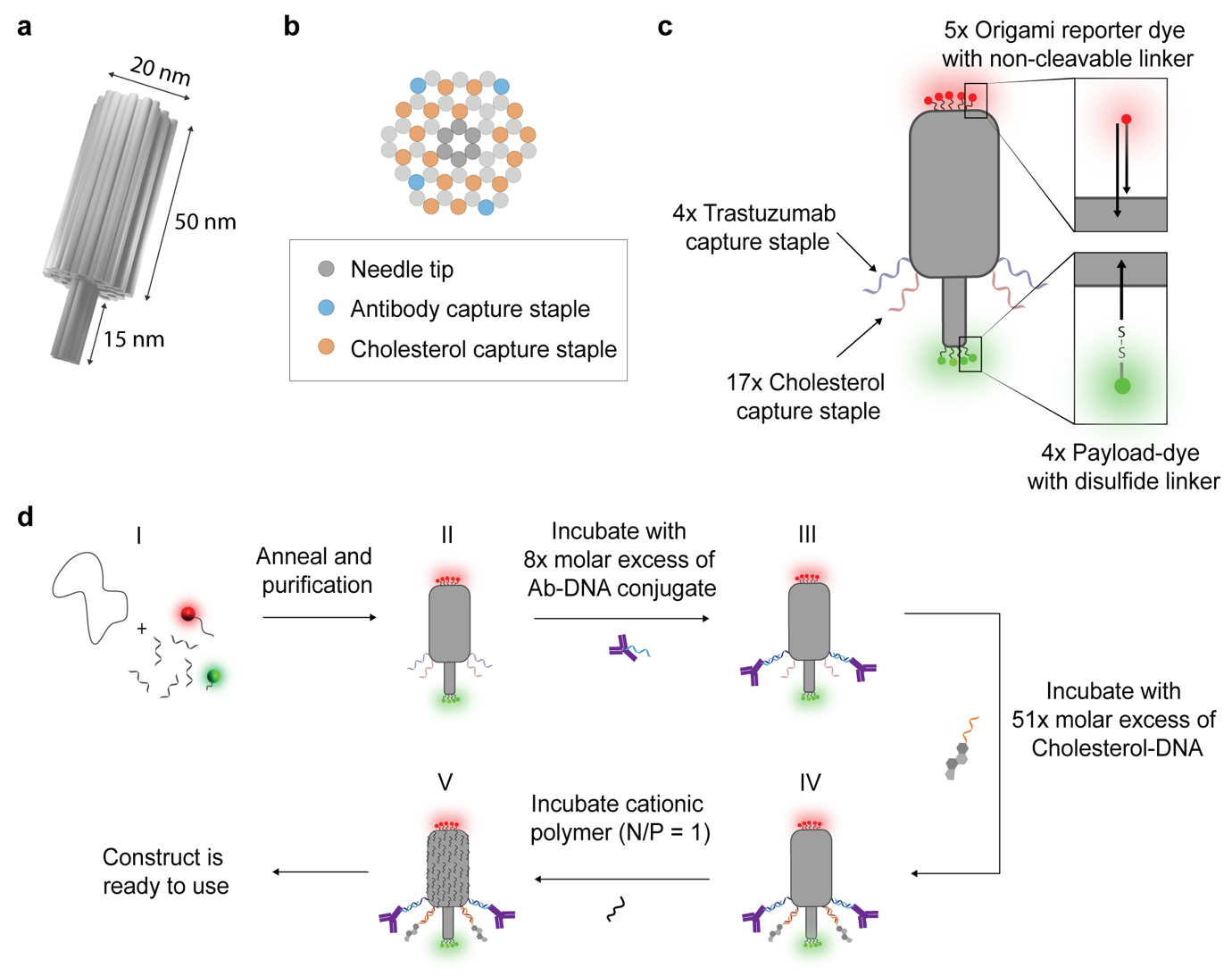


**Figure S4. Functionalization of the needle with trastuzumab-DNA conjugates.**

The origami needles were mixed with varying equivalence of trastuzumab-DNA conjugates per staple at room temperature. After, the samples were analyzed on an agarose gel. The gel conditions were 1% agarose with 12.5 mM MgCl2, 1x TAE, 80V, 45 mins, SYBR safe stain.


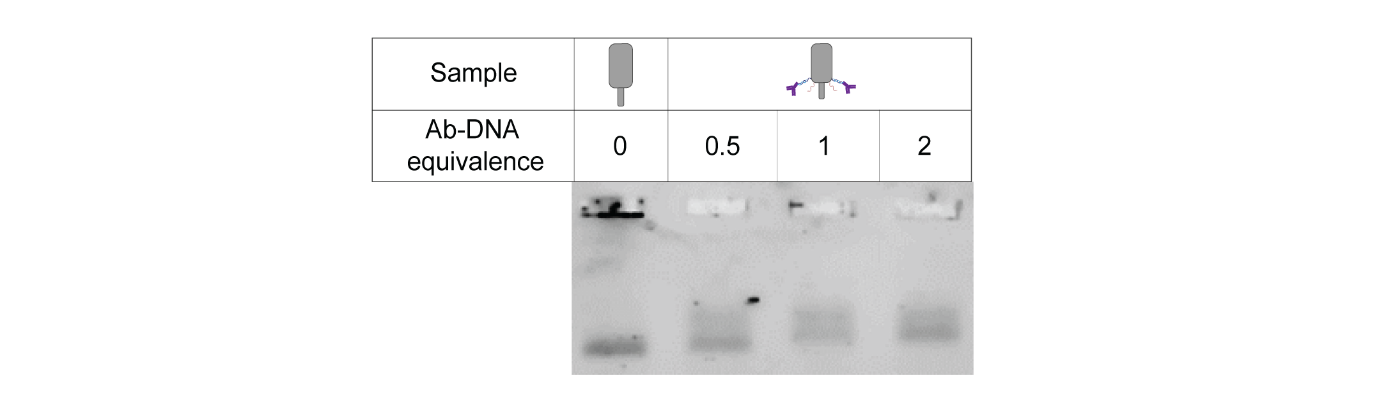


**Figure S5. TEM image of cholesterol-functionalized needles with liposomes.**

The liposomes were mixed with cholesterol-functionalized needles and visualized by TEM. (a) Full TEM image of the liposome-cholesterol-functionalized needle sample, mixed at a 1/100 dilution for 1 hour at room temperature. Magnification is 52,000x and scale bar is 100 nm. (b) The experiment was repeated using an adjusted liposome preparation protocol. The liposomes and cholesterol-functionalized needles were mixed at a 1:0.3 ratio for 1 hour at room temperature. Magnification is 15,000x and the scale bar is 200 nm. c-d) shows more images of using 15,000x magnification. The scale bar is 200 nm. Crop-outs of these images are used in Fig. 2f. e) In a control experiment liposomes were mixed with needles without cholesterol functionalization. This shows almost no liposome attachment to the TEM grid, suggesting the insertion of the needle drives the blotting of the membrane inserted needle in liposome onto the TEM grid.


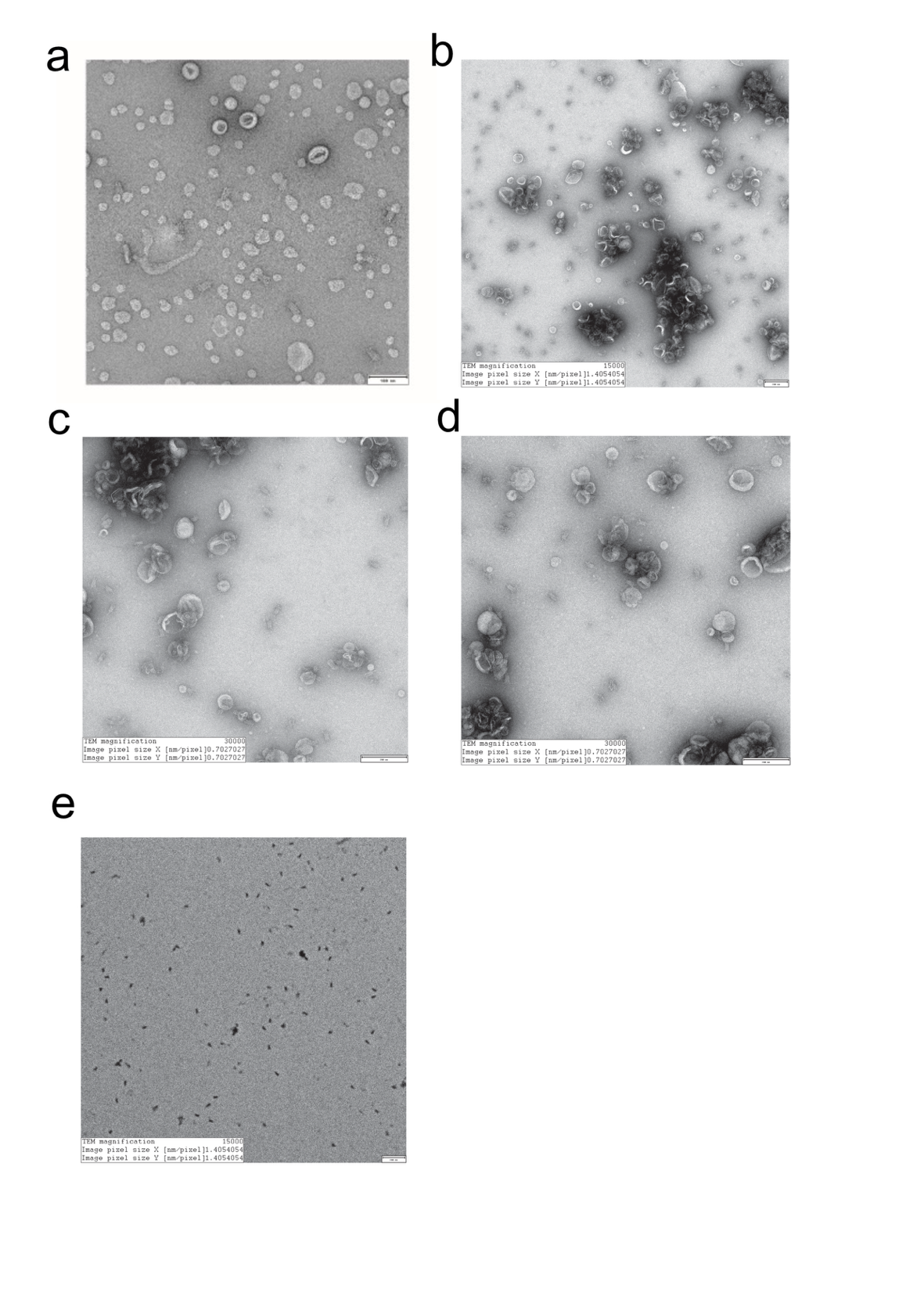


**Figure S6. Optimum PCD concentration for needle contruct protection.**

(a) Agrose gel electrophoresis of the needle origami mixed with different N/P ratios of PCD. The origami was purified into a lower salt buffer (50 mM HEPES, 0.8 mM MgCl_2_, 0.9 mM CaCl_2_, 200 mM NaCl, pH 7,4) before mixing with PCD. The gel conditions were 1% agarose with 12.5 mM MgCl_2_, 1x TAE, 90V, 35 minutes with SYBR safe stain. (b) TEM images of needle structures with the cationic polymer with and N/P ratio of 0, 0.5, 1 and 5. Magnification is 42,000x with a 200 nm scale bar.


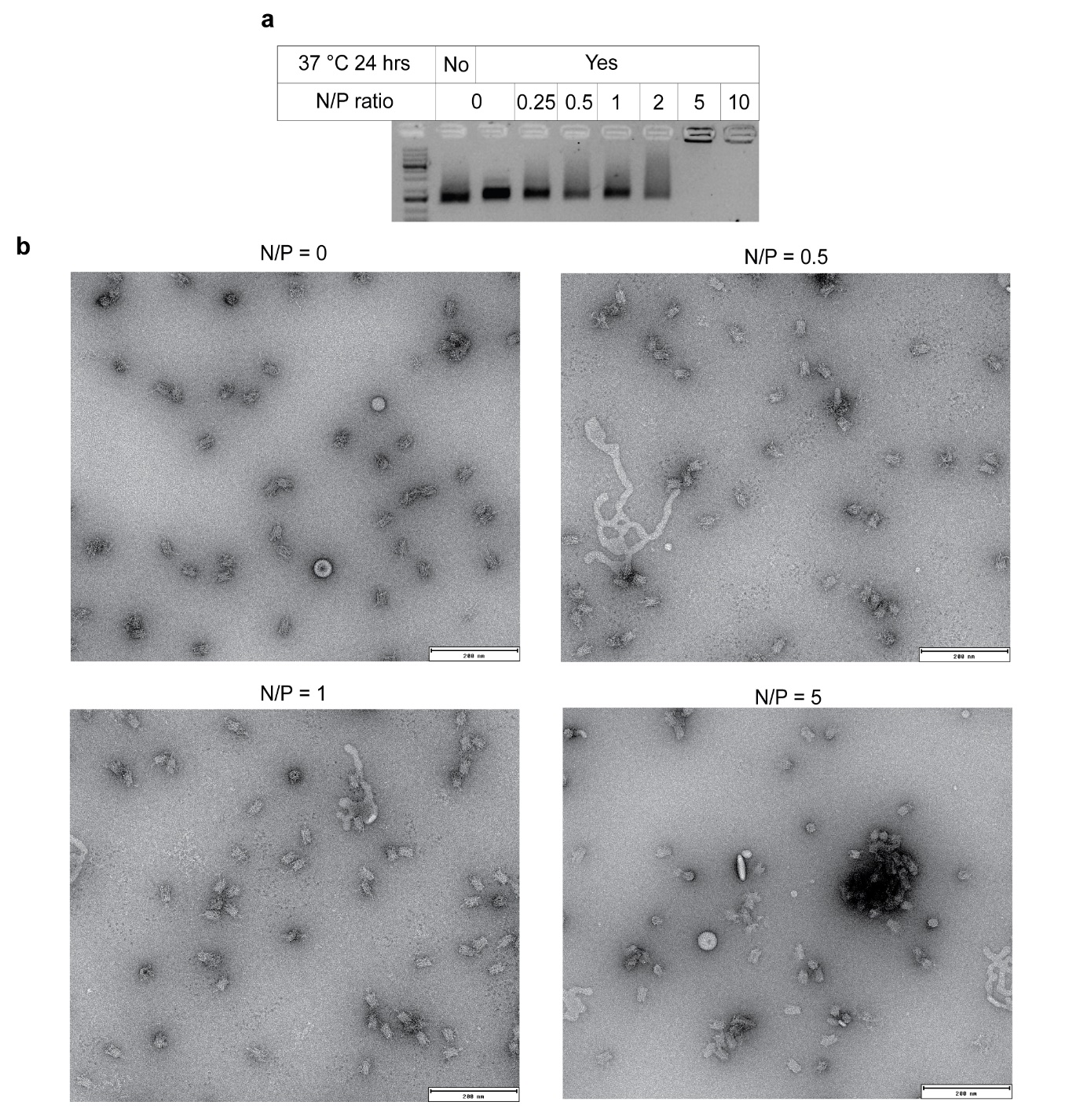


**Figure S7. Stability study of needle protected with PCD polymers in 10% FBS.**

(a) Agrose gel electrophoresis assessing the stability of the needle origami with PCD (N/P = 1) in 10% FBS. The origami was purified into a lower salt buffer (50 mM HEPES, 0.8 mM MgCl_2_, 0.9 mM CaCl_2_, 200 mM NaCl, pH 7,4) before mixing with 10% FBS at 37 °C. The gel conditions were 1% agarose with 6.25 mM MgCl_2_, 1x TAE, 80V, 35 minutes with SYBR safe stain. A crop-out of this gel was used in Fig 2g.


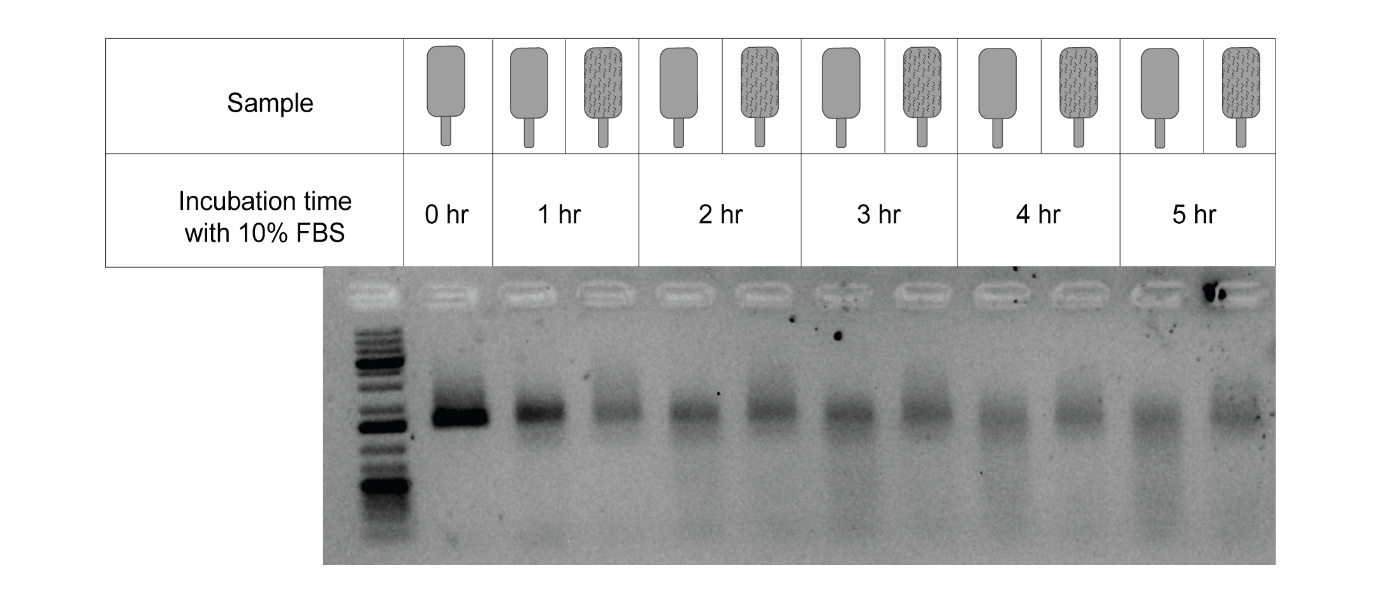


**Figure S8. Reaction scheme for synthesizing the payload-dye with the disulfide cleavable linker**

Amino-modified DNA staple strands was reacted with disuccinimidyl suberate and shaken at room temperature for 30 minutes. The produce was precipitated and HPLC purified before reacting with Cy3-NHS or SeTau647-NHS ester. The mixture was shaken at room temperature overnight before precipitation, purification (RP-HPLC) and lyophilization.


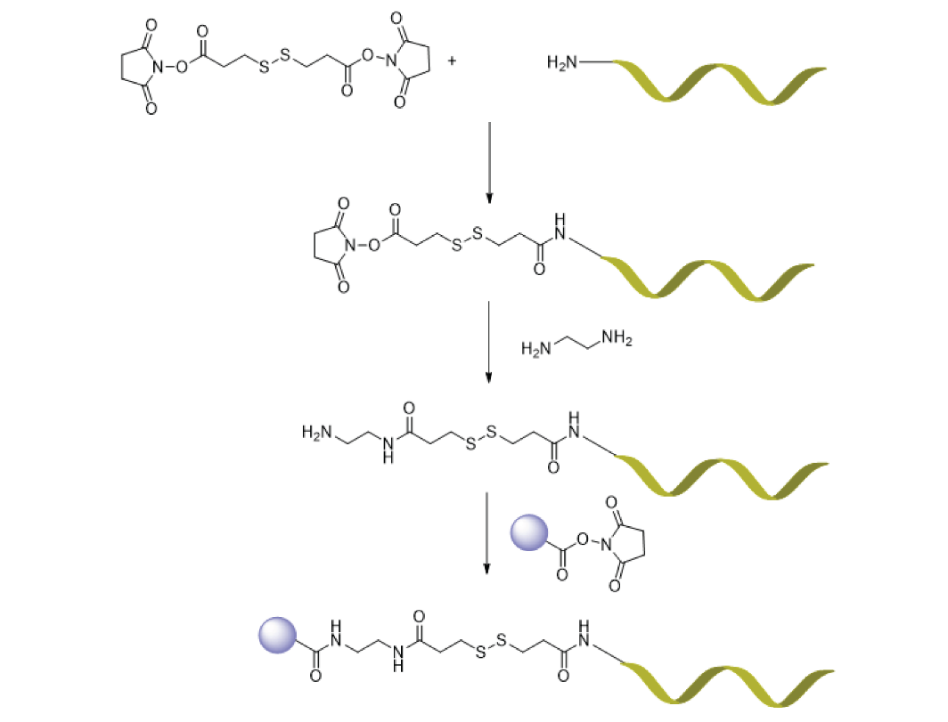


**Figure S9. Needle uptake studie using spinning disk confocal imaging.** Confocal imaging for uptake studies for five needle constructs and a ctrl with no added construct. The five construct are a) the full construct, b) the full construct without cholesterol, c) the full construct without antibodies, d) the needle construct with PCD, e) the needle construct unprotected. Which allow us to study the effect of antibodies, cholesterol and PCD protection by systematically leaving one out. From the quantitative images it is found that the full construct shows significant more uptake than the construct with no antibodies or cholesterol, indicating a clear targeting effect of having both present and showcasing that the needle structure indeed is capable for a targeted delivery of payload.

**
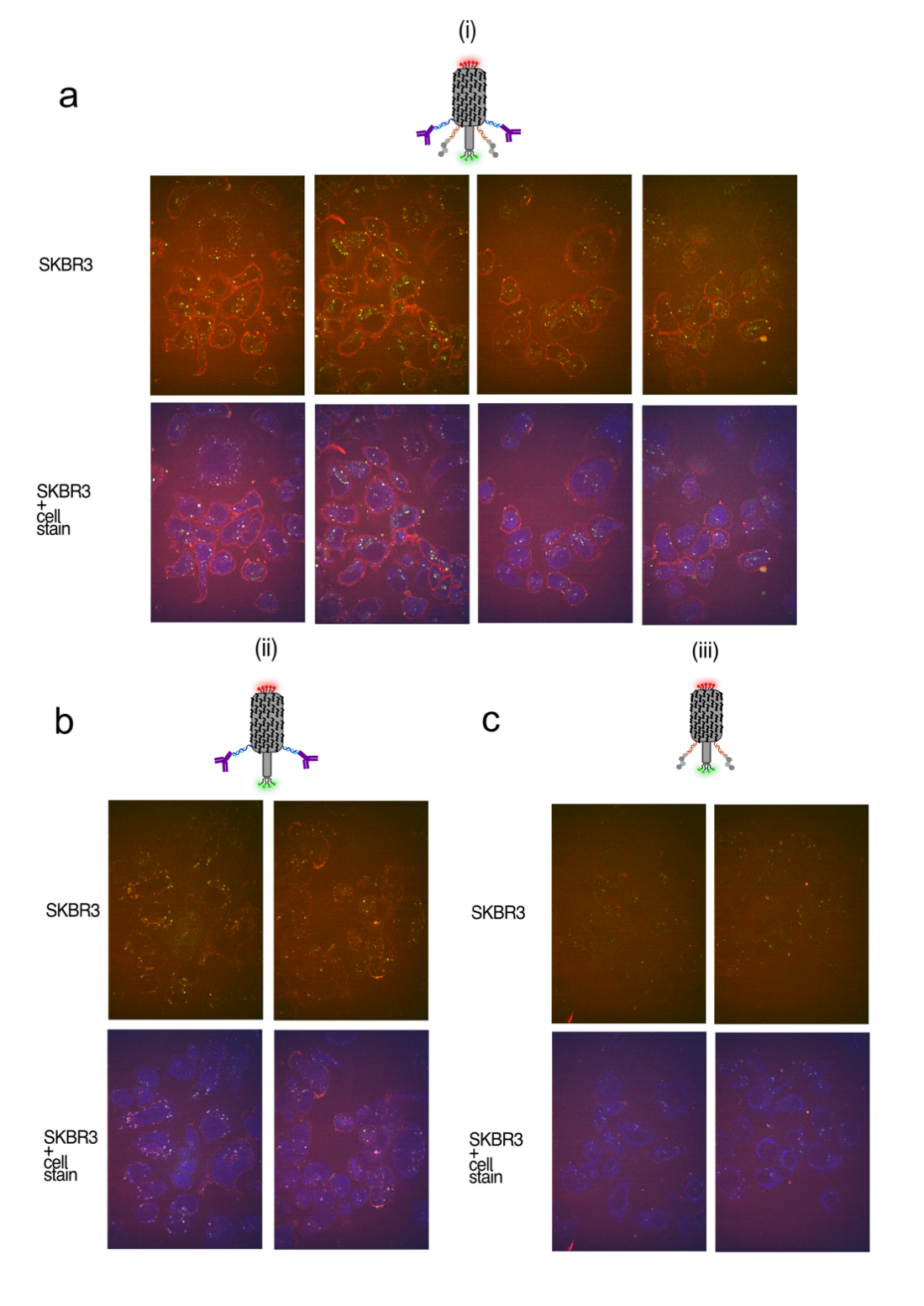
**

**
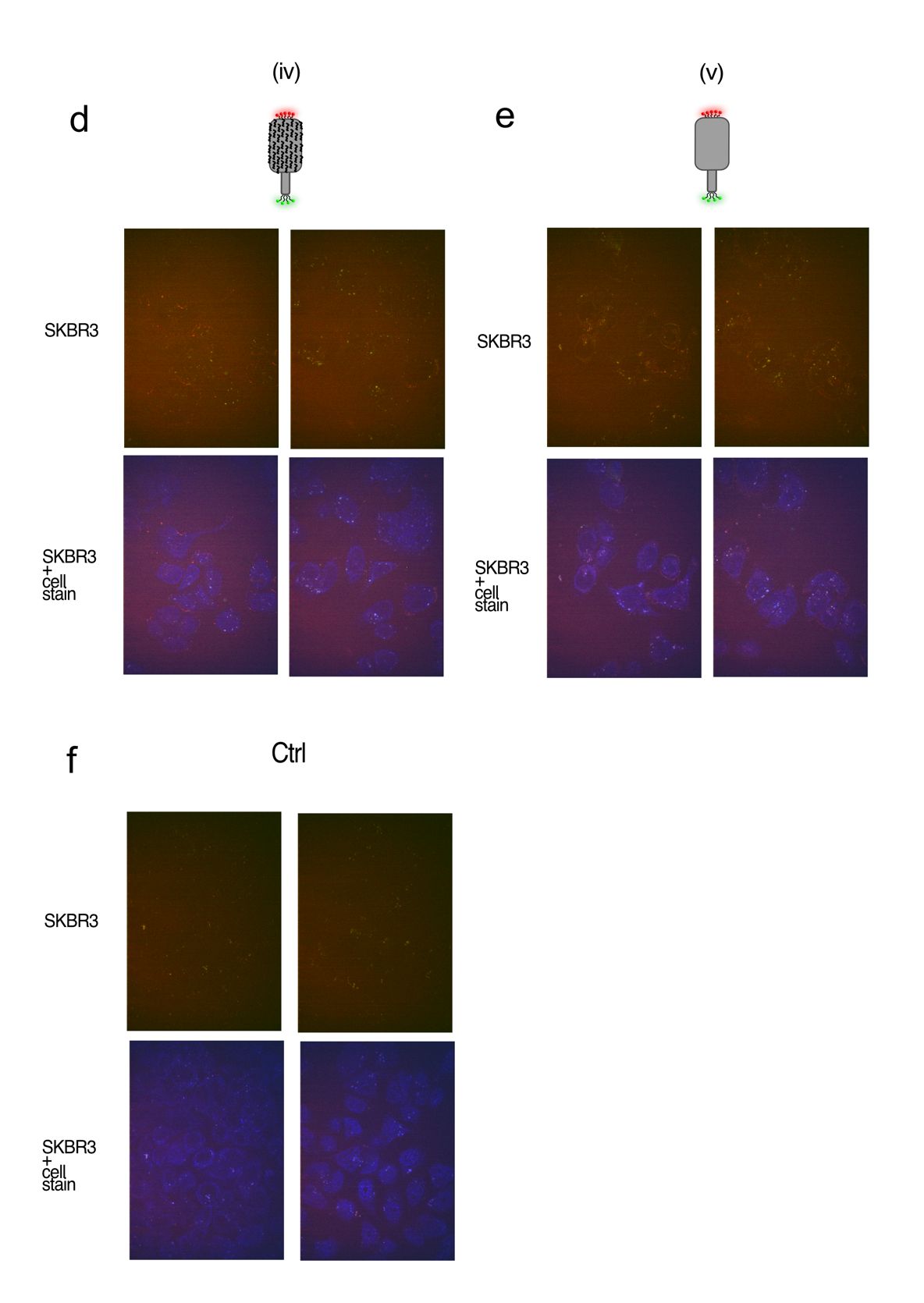
**

**Figure S10. Colorblind friendly images of spinning disk confocal images of SKR3**

Colorblind friendly images of the Main Figure 3b spinning disk confocal images of SKR3 cells systematically treated with five different needle constructs and control ; i) the full construct, ii) the full construct without cholesterol, iii) the full construct without Ab-DNA, iv) the needle construct with PCD, v) the needle construct unprotected and a untreated (UT) control with no construct. Here the colors were changed in FIJI using LOT, however, the contrast was kept the same as in Main Figure 3b.

**
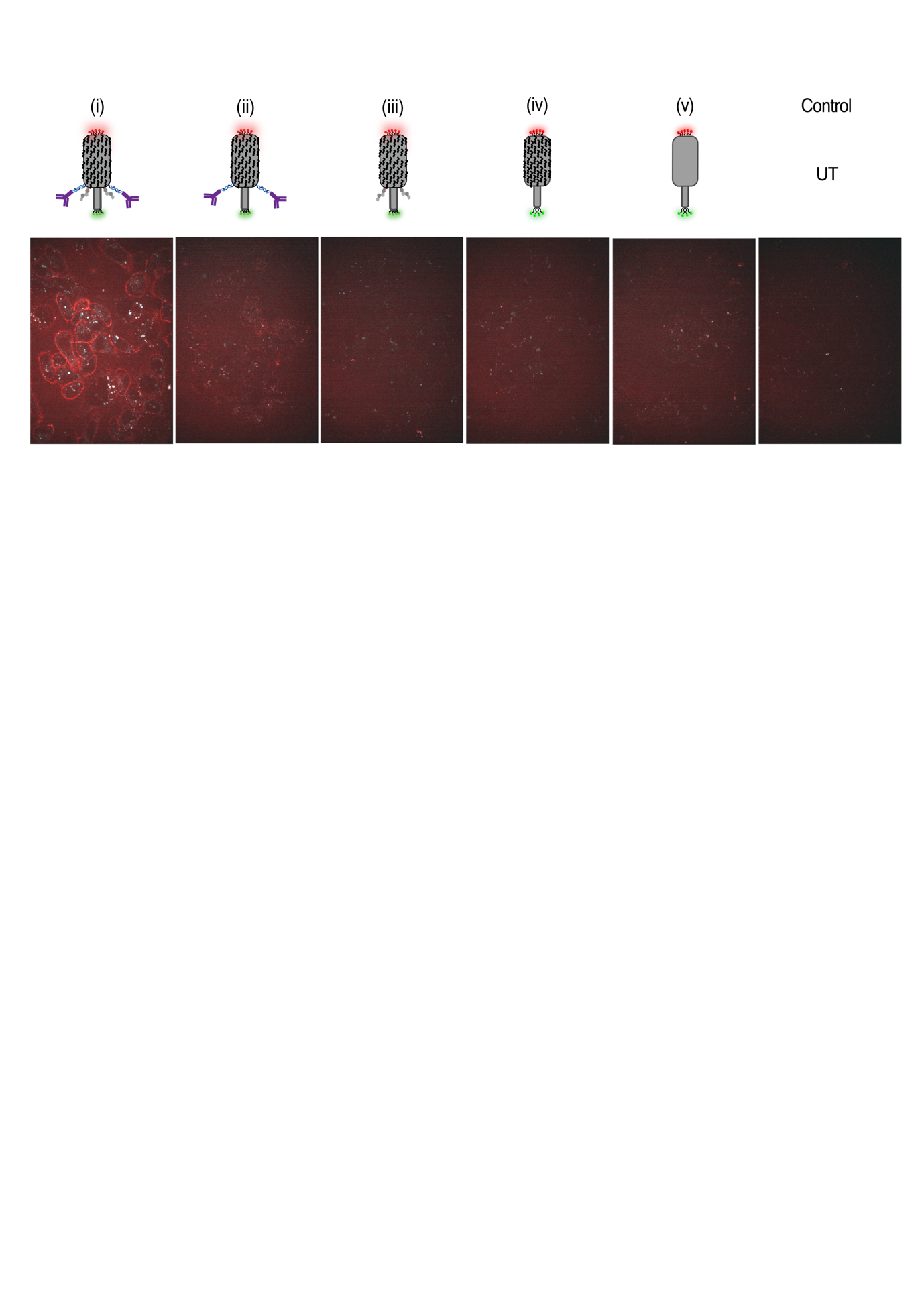
**

**Figure S11. Cellular uptake analysis of payload delivery from the needle.**

SKBR3 cells were imaged live using HILO microscopy with alternating laser excitation at 488 nm and 640 nm (100 ms exposure). Endosomes were fluorescently labeled (green) using CellLight™ Early Endosomes-GFP. The full needle construct carried a SeTau-647 payload on a cleavable disulfide linker (red) to enable quantification of endosomal uptake (Supplementary Fig. S11). Qualitative images (Supplementary Fig. S11a–b) show intracellular payload with some overlap with endosomes and a substantial nonoverlapping fraction consistent with cytosolic delivery. For quantitative colocalization, segmented payload and endosomes were analyzed in Fiji using Coloc2 plugin (Supplementary Fig. S11c): Pearson’s correlation coefficient was near zero (mean 0.047±0.036), with marked heterogeneity across ROIs. Manders’ coefficients indicated partial overlap (M1 =0.507±0.019; M2 =0.354±0.020). Together, these data support parallel endosomal uptake and substantial cytosolic delivery. All errors reported are the standard error of the mean (SEM).

**Figure S12. Single molecule tracking insights of cleavable and non-cleavable linked seTau.**

a) From SPT of needle structure with both cleavable and non-cleavable linked seTau payload we fitted the Mean-square displacement (MSD) within all trajectories to calculate the anomalous diffusion exponent (α) for each molecule. The α values are found to be highly significant larger for payload with cleavable linkers than non-cleavable linked payload, which might be due to delivery and not only membrane association. (significance tested with a two-sided Kolmogorov Smirnov test, P = 4.87×10^-10^). b) Mean-square displacement (MSD) and anomalous diffusion exponent (α) this we determine for each particle if it is fast (α>0.8), colocalizing with the cellular membrane and subsequently moving, or slow (α<0.3) colocalizing with the cellular membrane and subsequently moving. Moreover, molecules fount in membrane more than 10 frames and α<0.3 were classified as attached, and molecules in the membrane more than 10 frames, α<0.3 and subsequently moving were classified as temporarily fixed at the cell membrane. c) For quantifying the percentage of identified and tracked payload inside the cells vs. payload identified at the membrane of the cell, we calculated the percentage of fast-moving payload (α>0.8) and percentage of fast- and slow-moving payload (α<0.3) inside the cell compared to all identified payloads.

**
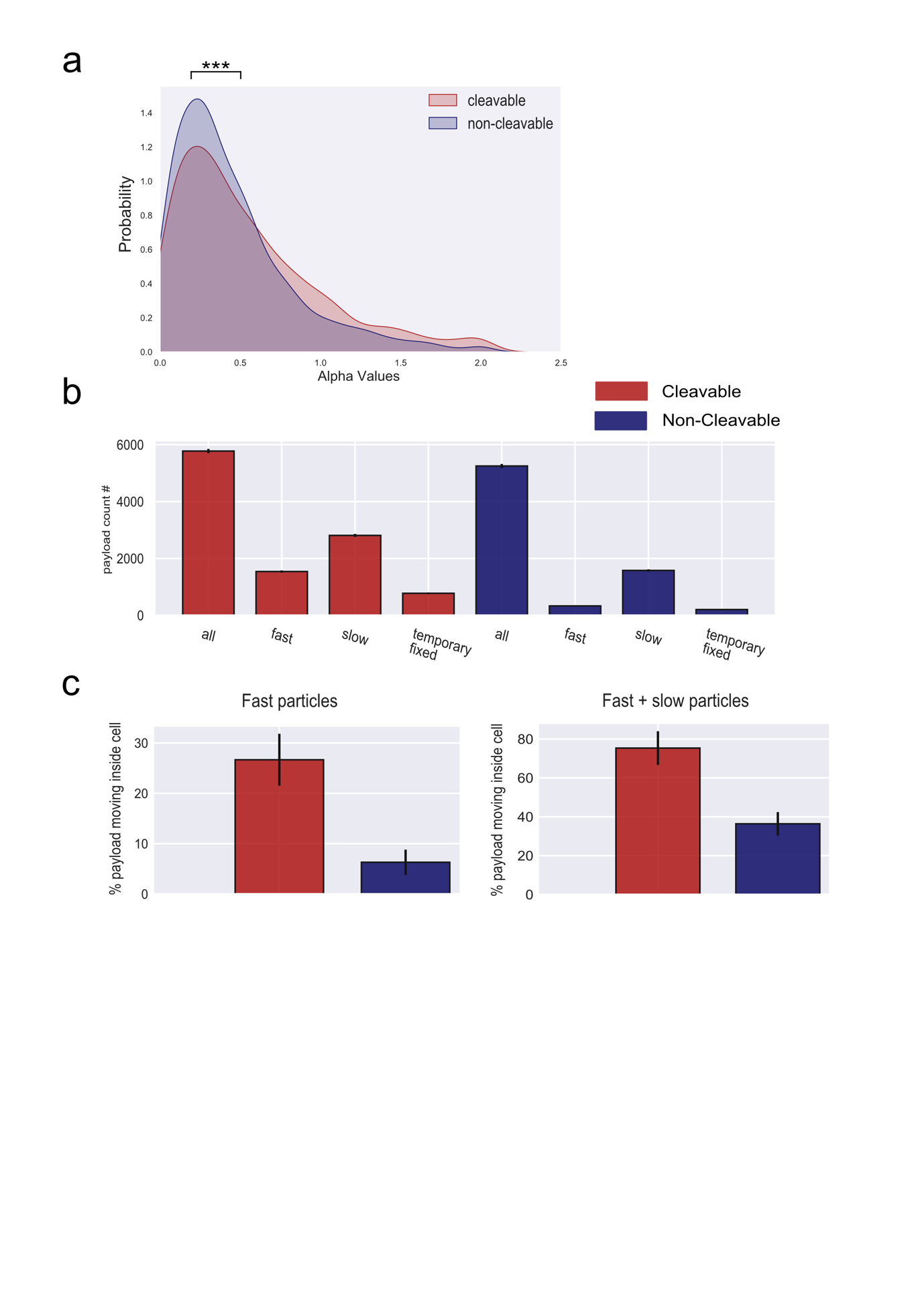
**

**Table 1. Table of core sequences for constructing DNA origami needle**

| **Name** | **Sequence** |
| --- | --- |
| 0[104] | TCACCGTTTGACCCAACGGAGTATAAGCTAAAATTCGCATTA |
| 0[153] | ATAAGTGGTTTGCCGTTTGCCTTACCAAGAGGTTTTTATTAATTTTAAA |
| 0[160] | CAGGCGGGTAACGGGGTCAGTGCCTTGAGTTTTGC |
| 0[181] | TAGCGGGGTAACAGACAGGAGTGTACTG |
| 0[202] | CCTCAAGGTTAATGTCATACATGGCTTTCTTGATA |
| 1[77] | AACCGCCGGCTGACCTTCATCGCTTGCCCTGACGAGCCATTC |
| 1[91] | GAGCCACGCGTAACACGAACTTACCACATCACCAGAGCAAGC |
| 1[119] | CAAGCCCCAAGAGGACAGATGCTTGAGATGGTTGCAAGCACC |
| 2[118] | CACAGACATTACAGGTAGAGTACTAATTTCAACTTAACTCAC |
| 3[91] | AGACGTTGAGGGGGTAAAAACTCCAGTTCCCTTATAAATCAA |
| 3[126] | GCTAAACTGCGAATCCAAATAAAACAGCATATTCCCACCAGA |
| 3[168] | GAGGCAGCCGCCGCCAGCATTCATAATCTCAGTAC |
| 3[182] | GATTGGCTGATGATTGCCCGTATAAACA |
| 4[90] | GGCTCCAACAGGTCAGGATTACTGACTATTCAGAACAGAACA |
| 4[139] | AAGGAATAACTTTCCCCACAAAACAATGCTTTTTTGTGAATA |
| 4[153] | GAAAGGAGTTTAACCTAATTT |
| 5[70] | TTAAACACCTTTAATTGTATCCCAGACCGGAAGCAAAAGCGG |
| 5[105] | GACAACAACCATGGTTTTCACCTGTATGCCTGCAAAAGAAGT |
| 5[140] | TATTAGCCCGTCGATCACCGTAGGGAAGTCTGTCCGCCAGTA |
| 5[168] | AAAATCACCGGAACACCAGAGGTCAGAC |
| 6[83] | GGCTTGCACCCTCAGAAGTTTGGTAGCT |
| 6[118] | TCCGCCCACGCATATCTGGAAGTTTCTTTTCCTTTTGATAAG |
| 6[125] | GTTTTCAGGAAACGTCACCAAATCAGATATAGACTGAAGTAC |
| 6[160] | GAATCAAATCTTTTGAGAATAAGTGACAGGAGGTTGTAATAAGTTTTTT |
| 8[132] | TAGCAAGGCCGGCTGCACAAGAAAAATAGGTATTAAACCACC |
| 9[84] | TAAAACATCATGAGGCAGCGAAAGACAGTATTCGGTCGCTGA |
| 9[133] | TGAATTAGAGGGTTACCCATGACTACAAAGGAAACCAGATAG |
| 10[111] | AAAGTACCCAGCGACTTTGAGACGAGGGGTAGTAGAAAGGTGGCATCAA |
| 10[125] | AAATTGCTACGACAAAAGGTAGATAAGTCCTGACTGAGAGTC |
| 12[90] | ATAGGCTACCCTCAAGGTTTAACCGATAGTTGCGCAAAAAAA |
| 12[132] | ATTTTGTAATAGGAGATATAAGGTCATAGTAGCGCGCTACAA |
| 12[139] | TAAGTTTTAGCAAATAAGACTTAGGTTGGAGACTACCTTTTT |
| 13[91] | CAGAACGTACCTTAGGGGTGCCAGTCGGGAAACCTTTTCTTT |
| 14[104] | TGTGAATAGTAGTAAAACCAGAAGATCGCACTCCACGGCGGA |
| 15[98] | AACGGAACAACATTAGCCCTCGAATTTTGTTGAAACCTTTAATTGCTTA |
| 15[140] | CGAGGAAGCATGATCGTAGAAGTGATAACATAATTACTAGAA |
| 16[90] | TTCAACTACGACGATAATAGTTAAACAGTTATAGTTATTTTT |
| 16[111] | TTGAGATGCGAGAGGCTTTAAATAGATAGGGTTGAAACTCAA |
| 18[104] | TTTGCCAAGTAAATATAGTTACACCCTCTACAGACAGGGAACCGAACTG |
| 18[146] | GAGATAAAACAGTTAGTACAATACCGTACACGGAACATATGG |
| 18[153] | TATCAGAAAGTCAGTACAGAGAAATAAAGGATTATACTTCTG |
| 19[105] | ATGACCATAAATGAGCCGGCCTTGCTGGATTTACA |
| 20[83] | CAGAAGCAACTCCAAAAGGAGGCTTGATGTACCGCAATACAC |
| 20[118] | TACAAAAATCAGGTCTTTAATGCGCGCCCAAGATTCACCAGT |
| 21[119] | TCCCAATAATAATTCATTTTCGTATAGCAAATTCATTAAAGG |
| 22[104] | AGGTCATTACGGTGACCGATACATCGGAGACTAAATACAAAG |
| 24[118] | TTATTCCATATAACACAAACAATTCGACGAGAGCCAGCAGCA |
| 24[132] | AAGATTAATTAAATCCTTTGCAAGAAACTGATTATAATAATG |
| 24[139] | TTAAATCTTTTATCACAAAATAGAAACGCATAAAAGAACACC |
| 26[146] | CCAATAGACAAGCATCATTCCAAGAACGATATCCCTTACCAT |
| 27[126] | CGCACTCATCGAGACAAGCAATGAAACCTCAGACTGCCCCCT |
| 28[104] | CGGGAGAAGCCTTTAGAGATCGACTTTTCTCATCTACTCAGG |
| 28[118] | CTGTAATGGTCATTACAGAGGTTATACCCCGGAATGGGATAG |
| 29[105] | GCTATCAACTTTTGACCAAAAACATTATACTAATATAGCAAC |
| 29[140] | ATCCTAAGTTTATCAACAATAAAGTAATGTAAATAGAAAATT |
| 30[104] | AACAAGAGAATCGAAAGATTGATTTGTAAATCATACAGGCGC |
| 31[112] | TAAATTGGCGAAACACCAACTTTGAACAACCGTGGGAACAAA |
| 32[125] | AGTACAACGTTAATCGTCGGATTCTCAGTAGGGCTAATATAA |
| 32[139] | ATAAGAGTAATTGAAAAGCCACCGGAATATAAGGCAATATAT |
| 33[91] | AAATGTGTAATGGGCCTCAGGGCAAAGCGGTCGACTTTCCTG |
| 34[104] | TTGACCGAGCGAGTAACAACCATTTTGTAAATATTTGGAGCA |
| 34[146] | CCAGTATGAATCGCCATATTTTTTTCGAAGACGACCGCGCCT |
| 34[153] | ATTCTTATTTACCATTGAGGGCACCGACCACCAGTGCAGCAC |
| 35[112] | GCCAGCTTTCCGGAATAAACAACGCTCAATCAATATTGACGG |
| 36[76] | AGGCTGCTGCCAAGCTTGCATGCCTGCAGCCATTCGAAACAC |
| 36[118] | GCTTCTGGGGTACCGAGCTACTTTTTCAGTTAAATAGTATGT |
| 37[105] | GATCCCCGTGCCGGAATTGGGAACGGTGATTTTCAAGGTGTA |
| 37[140] | TTTAGTTAATTTCACGACCGTAATACATAAGACACACACTGA |
| 38[139] | CAAAGAACGCGAGATCCGGCTCCTTATTTACCAGACGCCTGT |
| 38[154] | AATCCAATTAACTATAGAACTGACGCAATCGTCACCTCAGCGG |
| 39[119] | ATTAACCAACGAATTCGTAATCATGGTCGTGAGCTTAATCAT |
| 41[112] | GCTGATTGCCCTGAAATTGCGTTGCGCTCACTGCCGGCAACA |
| 41[126] | TAGCTTACCGAACAAGCAAGAGAATTGAATTAACTACAGGGA |
| 41[133] | GATTAAGTCCTTGAAAACATATATGTGAAATGGAATGATGAA |
| 42[104] | GAGTTGCTGAGACGCGCTTTCCTAATGAATAGCTGTCTAGAG |
| 42[111] | CCTGAGAAAGAATAGCCCGAAATCAATAGCTCACCGCCTGGC |
| 42[156] | TTAATTTTCCTTCTGTATTCATTTAACAAAACGCGCAGTGCTTTG |
| 43[140] | ACCTTGCCTTAGAAACGCTGAGGTCTGAGGTTATACGCAAGA |
| 44[90] | TGGAACACATCACTTGCCTGAGTAGAAGGTGTTGTCAAAATATTAGGAA |
| 45[112] | ACTATAAAAGAAGAACAGTACTAATAAGAAAAGATTCATCAG |
| 46[132] | TTTCAATACAATAAAACAGTACCTTTTAATATCAAAGCGCAT |
| 46[139] | TTATTCAACAAACATCAAGAAGAATTACAAATAGCAAGTAAG |
| 47[112] | TTGGCTCGGGAGAATACCTGACGGGAGAGTTAAGCGGATTTTAGCATTC |
| 48[104] | CACACGATATTAGTCTTTACCGAGAGTAATCTCCACGACAAT |
| 48[153] | AACGTCAGATGAATAAAACAGAGAATAAATTTTTTACAACTA |
| 49[91] | GAATGGCCCAGTAAGTCTGAAATGGATTTAATATCAACGAGA |
| 49[126] | AATTATTGAAGGGTTAGAACCTATCATCCATATTAGCACCCA |
| 49[133] | TGCACGTATACAGTCGGATTCGCCTGATAGGCGAACTGAACA |
| 51[105] | GTGCCACGCTGAATTAAACTGATAGCCCTAAAACATGCAACA |
| 51[140] | CAGATGAGCCAGTTCTGAATCTTTAGCGATCGATAAGCACCA |
| 51[147] | TGGCAATCATTTTGCGGAACACCGAACGTGAAGCCAGAACGC |
| 52[90] | AGCATCACCTTGCTATTTGAG |
| 52[104] | AATGAAAAGACTTTAGTTGATGAGCTGACATTAACCGGTTGT |
| 52[125] | AGGAGCGAACTCGTGTTGCTATTAGGCTTATCCGGTATTCTA |
| 18[63] | ATACATAACGCCAAATCATAAGGATAGC |
| 21[62] | TTGAATCATTGCATCAAAAAGAAGCGAA |
| 17[76] | CCCCTCAAATGCTTAAAATGTTTAGACTCCCTCGT |
| 49[83] | ATATTACCTCAATCTAAAAGGGACATTCCAGACAA |

**Table 2. Table of unfunctionalized antibody capture staples**

| **Name** | **Sequence** |
| --- | --- |
| 29[147] | TTTACGATTCCTTAAGCCGTTTTTATTTTCATC |
| 32[169] | AATTTAGGCAGAGGCAAACAACGCCAACATGT |
| 44[162] | TTTAACAATAATCGTCGC |
| 50[165] | TCCTGATTGTTTGAAATTGCGTAG |

**Table 3. Table of functionalized antibody capture staples**

| **Name** | **Sequence** |
| --- | --- |
| 29[147] | TTTACGATTCCTTAAGCCGTTTTTATTTTCATCAGTCGAAGAGCACTAGGTAGAG |
| 32[169] | AATTTAGGCAGAGGCAAACAACGCCAACATGTAGTCGAAGAGCACTAGGTAGAG |
| 44[162] | TTTAACAATAATCGTCGC AGTCGAAGAGCACTAGGTAGAG |
| 50[165] | TCCTGATTGTTTGAAATTGCGTAGAGTCGAAGAGCACTAGGTAGAG |

**Table 4. Table of unfunctionalized cholesterol capture staples**

| **Name** | **Sequence** |
| --- | --- |
| 8[169] | TTAGAGCCAGCAAAATTTGAGCCATTTGGGAA |
| 10[169] | GGCGACATTCAACCGAGCGCCAAAGACAAAAG |
| 12[169] | ATATAAAAGAAACGCAACATAAAGGTGGCAAC |
| 14[162] | ATACCCAAAATGTAAATG |
| 16[162] | AAGCCCTTTCAATAGTGA |
| 18[162] | GAGCGCTAAATCTTACCG |
| 20[165] | AATAGCAGCCTTAGGGTAATT |
| 22[165] | TCTTTCCAGAGCGTCAAAAATGAA |
| 24[165] | CCCGACTTGCGGCGCTAACGAGCG |
| 26[153] | ACCGCGCGAGGCGTTTTAGCGAACCT |
| 26[165] | GTAGGAATCATTCGTAATCAGTAG |
| 28[169] | CAATAATCGGCTGTCTGCATGTAGAAACCAAT |
| 30[169] | TTCAGCTAATGCAGAAGACAATAAACAACATG |
| 34[169] | CATATGCGTTATACAAAAAGCCTGTTTAGTAT |
| 36[169] | TAATGGTTTGAAATACTCTTCTGACCTAAATT |
| 40[162] | ATTTATCAAAATCATAGAAGAGTTTAAGAAAATAGCT |
| 48[165] | ATTTTCAGGTTTAATACCAAG |
| 52[165] | GAGTAACATTATTCATCAATATAA |

**Table 5. Table of functionalized cholesterol capture staples**

| **Name** | **Sequence** |
| --- | --- |
| 8[169] | TTTTT TTAGAGCCAGCAAAATTTGAGCCATTTGGGAA TGACAGGATTAGCAGAGCGAGG |
| 10[169] | TTTTT GGCGACATTCAACCGAGCGCCAAAGACAAAAG TGACAGGATTAGCAGAGCGAGG |
| 12[169] | TTTTT ATATAAAAGAAACGCAACATAAAGGTGGCAAC TGACAGGATTAGCAGAGCGAGG |
| 14[162] | TTTTT ATACCCAAAATGTAAATG TGACAGGATTAGCAGAGCGAGG |
| 16[162] | TTTTT AAGCCCTTTCAATAGTGA TGACAGGATTAGCAGAGCGAGG |
| 18[162] | TTTTT GAGCGCTAAATCTTACCG TGACAGGATTAGCAGAGCGAGG |
| 20[165] | TTTTT AATAGCAGCCTTAGGGTAATT TGACAGGATTAGCAGAGCGAGG |
| 22[165] | TTTTT TCTTTCCAGAGCGTCAAAAATGAA TGACAGGATTAGCAGAGCGAGG |
| 24[165] | TTTTT CCCGACTTGCGGCGCTAACGAGCG TGACAGGATTAGCAGAGCGAGG |
| 26[153] | TTTTT ACCGCGCGAGGCGTTTTAGCGAACCT TGACAGGATTAGCAGAGCGAGG |
| 26[165] | TTTTT GTAGGAATCATTCGTAATCAGTAG TGACAGGATTAGCAGAGCGAGG |
| 28[169] | TTTTT CAATAATCGGCTGTCTGCATGTAGAAACCAAT TGACAGGATTAGCAGAGCGAGG |
| 30[169] | TTTTT TTCAGCTAATGCAGAAGACAATAAACAACATG TGACAGGATTAGCAGAGCGAGG |
| 34[169] | TTTTT CATATGCGTTATACAAAAAGCCTGTTTAGTAT TGACAGGATTAGCAGAGCGAGG |
| 36[169] | TTTTT TAATGGTTTGAAATACTCTTCTGACCTAAATT TGACAGGATTAGCAGAGCGAGG |
| 40[162] | TTTTT ATTTATCAAAATCATAGAAGAGTTTAAGAAAATAGCT TGACAGGATTAGCAGAGCGAGG |
| 48[165] | TTTTT ATTTTCAGGTTTAATACCAAG TGACAGGATTAGCAGAGCGAGG |
| 52[165] | TTTTT GAGTAACATTATTCATCAATATAA TGACAGGATTAGCAGAGCGAGG |

**Table 6. Table of unfunctionalized reporter-dye capture staples at the needle base**

| **Name** | **Sequence** |
| --- | --- |
| 25[84] | TTCATTTGGGGCGCTCCCAATACTAAAGTTTTGCG |
| 29[84] | ATTTTTGATTTCAACGCAAGGATAAAAACGGAGAG |
| 37[56] | AAAACGACGGCCAGGCAACTGGAATAAGAAGAGTA |
| 45[70] | TTGATTAGTAATAAAGAGTCCTTACCAGAATGCAG |
| 53[84] | GATTTAGAAGTATTAATCTAACACCGCCTCGCCAT |

**Table 7. Table of functionalized reporter-dye capture staples at the needle base**

| **Name** | **Sequence** |
| --- | --- |
| 25[84] | TTCATTTGGGGCGCTCCCAATACTAAAGTTTTGCG TGGCTGTGGTGTGCGAGTAACT |
| 29[84] | ATTTTTGATTTCAACGCAAGGATAAAAACGGAGAG TGGCTGTGGTGTGCGAGTAACT |
| 53[84] | AAAACGACGGCCAGGCAACTGGAATAAGAAGAGTA TGGCTGTGGTGTGCGAGTAACT |
| 45[70] | TTGATTAGTAATAAAGAGTCCTTACCAGAATGCAG TGGCTGTGGTGTGCGAGTAACT |
| 37[56] | GATTTAGAAGTATTAATCTAACACCGCCTCGCCAT TGGCTGTGGTGTGCGAGTAACT |

**Table 8. Table of unfunctionalized payload-dye strands at the needle apex**

| **Name** | **Sequence** |
| --- | --- |
| 0[195] | AGAAGGAACCACCGGAACCGCAGAGCCGTTCACAA |
| 3[203] | ACAAATAGTAAGCGCCCCCTGCCTATTTAAGAGGCAGAGCCG |
| 4[195] | CCACCAGAACCACCCAGAGCCTTAGGAT |
| 4[216] | GAGCCACCACCCTCCTCCCTCTGAGACT |

**Table 9. Table of functionalized payload-dye strands at the needle apex**

Here Cy3 or SeTau647N was used as the fluorophore.

| **Name** | **Sequence** |
| --- | --- |
| 0[195] | Fluorophore-S-S-AGAAGGAACCACCGGAACCGCAGAGCCGTTCACAA |
| 3[203] | Fluorophore-S-S-ACAAATAGTAAGCGCCCCCTGCCTATTTAAGAGGCAGAGCCG |
| 4[195] | Fluorophore-S-S-CCACCAGAACCACCCAGAGCCTTAGGAT |
| 4[216] | Fluorophore-S-S-GAGCCACCACCCTCCTCCCTCTGAGACT |

**Table 10. Table of other sequences**

Here, Cy5 and SeTau647N was used as the fluorophore.

| **Name** | **Sequence** |
| --- | --- |
| Antibody strand | /C6NH2/CTC TAC CTA GTG CTC TTC GACT |
| Cholesterol strand | CCT CGC TCT GCT AAT CCT GTC A - Cholesterol |
| Reporter-dye fluorophore | /5AmMC6/AGTTACTCGCACACCACAGCCA |
